# Radiation-Induced Lymphopenia is a Causal Mediator of Survival After Chemoradiation Therapy for Esophagus Cancer

**DOI:** 10.1016/j.adro.2024.101579

**Published:** 2024-07-26

**Authors:** Yiqing Chen, Yan Chu, Peter S.N. van Rossum, Clemens Grassberger, Steven H. Lin, Radhe Mohan, Brian P. Hobbs

**Affiliations:** a Department of Biostatistics and Data Science, University of Texas Health Science Center, Houston, Texas; b School of Biomedical Informatics, University of Texas Health Science Center, Houston, Texas; c Department of Radiation Oncology, The University of Texas MD Anderson Cancer Center, Houston, Texas; d Department of Radiation Oncology, Amsterdam UMC, Amsterdam, The Netherlands; e Department of Radiation Oncology, Massachusetts General Hospital, Boston, Massachusetts; f Department of Radiation Physics, The University of Texas MD Anderson Cancer Center, Houston, Texas; g Department of Population Health, The University of Austin Dell Medical School, Austin, Texas

## Abstract

**Purpose:**

Radiation-induced lymphopenia (RIL) is common during chemoradiation therapy. Severe lymphopenia is associated with reduced survival. Proton beam therapy (PBT), with its substantially more compact dose distributions, spares circulating lymphocytes and immune organs at risk to a greater extent than photon therapy. Recent studies comparing PBT to photon radiation therapy, specifically intensity-modulated radiation therapy (IMRT) for esophageal cancer (EC), showed that the incidence of grade 4 RIL (G4RIL) is significantly reduced among patients receiving PBT for EC. However, whether the extent of this reduction has a direct causative link with improved survival is unknown. This study applies causal mediation analysis to answer this question.

**Methods and Materials:**

We retrospectively assessed 734 patients treated with concurrent chemoradiation therapy for biopsy-proven EC from 2004 to 2017. To address the potential for bias in the choice of radiation modality, propensity score analysis was used to evaluate and reduce imbalances between the PBT and IMRT cohorts. Causal mediation analysis was applied to decompose the total effect of radiation modality on overall survival (OS) into indirect (mediated through G4RIL) and direct effects.

**Results:**

We found that PBT was associated with a significantly lower incidence of G4RIL and prolonged OS compared with IMRT (odds ratio, 0.41; 95% CI, 0.28-0.60; *P* < .001). In the propensity-matched cohort of 506 patients (253 PBT, 253 IMRT), G4RIL risk reduction with PBT versus IMRT translated into a 5% reduction in the relative rate of death (*P* = .032). Mediation of G4RIL explained ∼14.5% of the difference in OS.

**Conclusions:**

G4RIL was found to mediate survival; however, a statistically significant direct effect of PBT on survival was not observed. In other words, the statistical significance of survival benefit from protons over photons in this EC cohort was lost in the absence of G4RIL risk reduction.

## Introduction

Esophageal cancer (EC) is the eighth most common type of cancer worldwide, ranking sixth in terms of overall mortality.^1^ With a 5-year relative survival rate of only about 20% in the United States, EC is one of the most aggressive malignancies.^2^ Radiation therapy (RT) is indispensable in the management of EC. However, radiation-induced injury to normal tissues such as the heart, lungs, esophagus, and hematopoietic system diminishes the quality of life and adversely affects survival.

Lymphocytes are among the most radiosensitive cells in the body and are key players in antitumor immunity. Varying degrees of lymphopenia are common during chemoRT. In EC, severe lymphopenia has been linked with poorer prognosis and worse overall survival (OS).^3^

The physical properties of protons used in proton beam therapy (PBT) facilitate increased tumor conformality and significantly reduce the dose to normal tissues, including immune organs at risk and circulating lymphocytes outside the target volume. Recent comparative studies have demonstrated the protective effects of PBT over photon-based RT with regard to severe radiation-induced lymphopenia (RIL) in patients with EC, particularly those with tumors of the lower esophagus.^4^^,^^5^ In a retrospective study, proton-based RT was associated with a significantly reduced incidence of grade 4 RIL (G4RIL) compared with photon-based RT during chemoRT for EC.^6^ Furthermore, the study by Zhu et al^7^ corroborated the impact of G4RIL on clinical outcomes, indicating that severe lymphopenia is a strong predictor of poor survival in EC patients. Ample evidence also suggests that PBT for EC leads to prolonged OS relative to intensity-modulated (photon) RT (IMRT).^3^^,^^8^ Wang et al^9^ supported the association between lymphopenia and reduced survival rates, which highlights that lymphopenia can be attributed to advanced disease stages and higher radiation doses, which in turn affects patient prognosis negatively.

However, whether any part of this observed survival advantage of PBT is attributable to reductions in the relative risk of G4RIL is unknown. This retrospective study was devised to test the hypothesis that G4RIL is not just an associative variable but a causal mediator of OS. Formal survival mediation analysis^10^, ^11^, ^12^ was applied to estimate the integrated causal relationships between radiation modality (PBT vs IMRT) and OS when decomposed into a natural (pure) direct effect (NDE) and natural indirect effect (NIE) mediated by the risk of severe RIL.

## Methods and Materials

### Study patients and inclusion criteria

We retrospectively reviewed records of 734 consecutive patients diagnosed with biopsy-proven EC and treated with concurrent chemoRT from January 2004 to November 2017 at a single tertiary care cancer center. This study was approved by the appropriate institutional review board with a waiver of the requirement for informed consent. The study included patients with (1) overall stage I, II, or III disease; (2) treatment consisting of either PBT with passively scattered proton therapy (PSPT) or IMRT; (3) planned median radiation dose of 50.4 Gy; (4) histologic diagnosis of adenocarcinoma or squamous cell carcinoma; and (5) availability of absolute lymphocyte counts at baseline and at least 3 weekly absolute lymphocyte count measurements during RT. Patients with a history of hematologic malignancy, endomucosal resection before chemoRT, or missing baseline blood sample records were excluded.

### Statistical analysis

Patient demographic and disease characteristics were compared between RT modalities (PBT vs IMRT). Categorical variables were summarized as frequencies and percentages, and continuous variables were summarized as means and SDs. *T* tests for continuous variables and X^2^ tests for categorical variables were used as appropriate to examine differences between the 2 radiation modalities. Logistic regression was used to estimate the odds of G4RIL by radiation modality, which were compared based on Wald's test. OS was compared by radiation modality and G4RIL using log-rank tests. All tests were 2-sided and used a threshold of .05 to indicate statistical significance. All analyses were performed in R v.4.0.2 (R Foundation for Statistical Computing).

### Propensity score analysis

To address potential bias in the process of selecting patients for each radiation modality, propensity score analysis was used to evaluate and reduce imbalances between the PBT and IMRT cohorts by applying the well-established R package “*MatchIt.*”^12^ Univariate analyses were conducted to assess potential confounders associated with both OS (Cox proportional hazards regression) and RT modality (logistic regression) observed at baseline. This was followed by multiple logistic regression with modality assignment as the dependent variable and significant baseline clinical characteristics identified from univariate analysis as independent variables. A final propensity score model was determined through backward stepwise model selection, with age and surgical status mandatorily included as fixed variables. The estimated probability of receiving PBT was calculated for each patient and served as the propensity score in subsequent analyses. Statistical analyses addressed selection bias in 2 steps. Radiation modalities were compared in a subset of patients matched 1:1 from the propensity score model using the optimal method, which ensures that the total sum of absolute pairwise distances is minimized within the matched sample. All regression models comparing G4RIL and OS were estimated from the matched cohort and adjusted for the propensity score, which was included as a covariate.

### Mediation analysis

An empirical method for estimating the causal mechanisms by which survival differences between treatment interventions are mediated by intermediate outcomes was initially proposed by T. VanderWeele.^12^, ^13^, ^14^ Regression-based causal mediation analysis was used for the matched cohort to evaluate the possibility that G4RIL is a causal mediator of OS. Mediation analysis was adjusted for the propensity score to control selection bias. To construct the mediation model, multivariate logistic regression was used to measure the effect of radiation modality on the mediator G4RIL. The corresponding odds ratios (ORs), 95% CIs, and *P* values are reported. Weibull accelerated failure time regression with shape parameter = 1 was then applied for survival analysis to model the conjoint effects of radiation modality and G4RIL on OS. The logistic regression model and Weibull accelerated failure time model were integrated for mediation analysis using the R package “*regmedint.*”^15^ The mediation model decomposes the total treatment effect (TE) hazard ratio (HR) for OS into a product of the NDE times the NIE mediated by G4RIL, ie, TE = NIE × NDE. Estimates of TE, NDE, and NIE are reported as HRs with corresponding 95% CIs and *P* values.

The HR for the NIE of PBT versus IMRT was converted into a median survival difference as follows. The marginal hazard rate of OS for patients receiving IMRT was estimated using the Weibull model. This hazard rate was multiplied by the point and interval estimates of NIE. Then, median survival was computed from exponential distributions with scaled hazard rates and compared with the marginal estimate for IMRT. The proportion of the total effect mediated by G4RIL (PM) was defined by the following equation^15^:

$$PM=\frac{\exp\left(NDE\right)\cdot \left[\exp\left(NIE\right)-1\right]}{\exp\left(NDE\right)\cdot \exp\left(NIE\right)-1}$$

### Surgical subgroup analysis

Considering that surgical resection after chemoRT is an established clinical prognostic factor associated with lower incidence of G4RIL and improved OS, mediation analysis was further conducted stratifying by whether patients underwent surgery after chemoRT or not.

## Results

### Patient characteristics and propensity score analysis

Patient and disease characteristics are shown in Table 1 for the overall study population (N = 734) and by radiation modality (IMRT n = 469 and PBT n = 265). A significantly higher incidence of G4RIL was observed in the IMRT arm (45.2% vs 22.6% PBT; *P* < .001). Propensity score analysis identified 7 significantly imbalanced factors: age, Eastern Cooperative Oncology Group performance status at trial entry, number of concurrent chemotherapy cycles, log-scaled planning target volume, Barrett's syndrome, tumor location, and overall clinical disease stage. Matching patients on these potential confounders reduced the analysis data set used for modality comparison and mediation analysis to 506 patients (IMRT n = 253 and PBT n = 253) (Figure E1). The distributions of patient and disease characteristics in the matched cohort are summarized in Table 2. Subsequent analyses were performed in the matched cohort only.

**Table 1:** Patient and disease characteristics for the 734 esophageal cancer patients included in the study

Characteristic	Total (N = 734)	IMRT (n = 469)	PBT (n = 265)	*P* value
Age, y				
Mean, SD	63.1, 10.7	61.7, 10.6	65.7, 10.3	<.001
(Min-max)	(20-92)	(20-86)	(26-92)	
Sex, no. (%)				
Female	110 (15.0)	72 (15.4)	38 (14.3)	.79
Male	624 (85.0)	397 (84.6)	227 (85.7)	
Baseline ALC, × 10^3^/μL				
Mean, SD	1.6, 0.6	1.7, 0.7	1.6, 0.6	.432
(Min-max)	(0.32-6.50)	(0.35-6.50)	(0.32-4.38)	
PTV, cm^3^				
Mean, SD	612.3, 279.3	661.5, 293.3	525.4, 228.1	<.001
(Min-max)	(92.8-2283.1)	(117.6-2283.1)	(92.8-1727.2)	
ECOG status at trial entry, no. (%)				
1 and 2	254 (34.6)	157 (33.5)	97 (36.6)	.438
0	480 (65.4)	312 (66.5)	168 (63.4)	
Tumor location, no. (%)				
Upper-middle	105 (14.3)	70 (14.9)	35 (13.2)	.60
Lower	629 (85.7)	399 (85.1)	230 (86.8)	
Disease stage, no. (%)				
I	41 (5.6)	24 (5.1)	17 (6.4)	.65
II	236 (32.2)	155 (33.0)	81 (30.6)	
III	457 (62.3)	290 (61.8)	167 (63.0)	
No. of concurrent chemotherapy cycles				
Mean, SD	5.0, 0.6	4.9, 0.7	5.0, 0.6	.008
(Min-max)	(1-7)	(1-6)	(2-7)	
Barrett's syndrome				
No	685 (93.3)	434 (92.5)	251 (94.7)	.326
Yes	49 (6.7)	35 (7.5)	14 (5.3)	
G4RIL, no. (%)				
No	462 (62.9)	257 (54.8)	205 (77.4)	<.001
Yes	272 (37.1)	212 (45.2)	60 (22.6)	
Histology, no. (%)				
Adenocarcinoma	617 (84.1)	397 (84.6)	220 (83.0)	.64
SCC	117 (15.9)	72 (15.4)	45 (17.0)	
If patient received surgery, no. (%)				
No	335 (45.6)	205 (43.7)	130 (49.1)	.187
Yes	399 (54.4)	264 (56.3)	135 (50.9)	

*Abbreviations*: ALC = absolute lymphocyte count; ECOG = Eastern Cooperative Oncology Group performance status; G4RIL = grade 4 radiation-induced lymphopenia; IMRT = intensity-modulated (photon) radiation therapy; PBT = proton beam therapy; PTV = planning target volume; SCC = squamous cell carcinoma.

**Table 2:** Patient and disease characteristics for the 506 patients in the propensity-matched cohort

Characteristic	Total (N = 506)	IMRT (n = 253)	PBT (n = 253)	*P* value
Age, y				
Mean, SD	65.0, 9.9	64.7, 9.5	65.3, 10.2	.460
(Min-max)	(26-91)	(27-86)	(26-91)	
Sex, no. (%)				
Female	91 (18.0)	55 (21.7)	36 (14.2)	.037
Male	415 (82.0)		217 (85.8)	
Baseline ALC, × 10^3^/μL				
Mean, SD	1.6 (0.6)	1.7 (0.6)	1.6 (0.6)	.301
(Min-max)	(0.32-4.38)	(0.5-3.53)	(0.32-4.38)	
PTV, cm^3^				
Mean, SD	548.2, 235.4	562.5, 244.2	533.9, 225.9	.172
(Min-max)	(103.8-1757.8)	(117.6-1757.8)	(103.8-1727.2)	
ECOG status at trial entry, no. (%)				
1 and 2	189 (37.4)	95 (37.5)	94 (37.2)	1.000
0	317 (62.6)	158 (62.5)	159 (62.8)	
Tumor location, no. (%)				
Upper-middle	72 (14.2)	39 (15.4)	33 (13.0)	.525
Lower	434 (85.8)	214 (84.6)	220 (87.0)	
Disease stage, no. (%)				
I	32 (6.3)	16 (6.3)	16 (6.3)	.981
II	150 (29.6)	74 (29.2)	76 (30.0)	
III	324 (64.0)	163 (64.4)	161 (63.6)	
No. of concurrent chemotherapy cycles				
Mean, SD	5.0, 0.6	5.0, 0.6	5.0, 0.6	.303
(Min-max)	(1-7)	(1-6)	(2-7)	
Barrett's syndrome, no. (%)				
No	483 (95.5)	243 (96.0)	240 (94.9)	.669
Yes	23 (4.5)	10 (4.0)	13 (5.1)	
G4RIL, no. (%)				
No	342 (67.6)	147 (58.1)	195 (77.1)	<.001
Yes	164 (32.4)	106 (41.9)	58 (22.9)	
Histology, no. (%)				
Adenocarcinoma	419 (82.8)	208 (82.2)	211 (83.4)	.814
SCC	87 (17.2)	45 (17.8)	42 (16.6)	
If patient received surgery, no. (%)				
No	235 (46.4)	114 (45.1)	121 (47.8)	.593
Yes	271 (53.6)	139 (54.9)	132 (52.2)	

*Abbreviations*: ALC = absolute lymphocyte count; ECOG = Eastern Cooperative Oncology Group performance status; G4RIL = grade 4 radiation-induced lymphopenia; IMRT = intensity-modulated (photon) radiation therapy; PBT = proton beam therapy; PTV = planning target volume; SCC = squamous cell carcinoma.

### Comparison of RIL by radiation modality

In univariate analysis, propensity-matched patients (Table 2) who received PBT were associated with a significantly lower incidence of G4RIL than patients receiving IMRT (OR, 0.41; 95% CI, 0.28-0.60; *P* < .001) (Table 3). This association remained significant in multiple regression analysis (OR, 0.35; 95% CI, 0.22-0.53; *P* < .001).

**Table 3:** Odds ratio of grade 4 radiation-induced lymphopenia development among the propensity-matched cohort (N = 506)

Covariate	Value or n (%)		OR (95% CI)	OR (95% CI)
	No	Yes	(univariable)	(multivariable)
Age, y				
Mean (SD)	64.2 (9.9)	66.8 (9.5)	1.03 (1.01-1.05; *P =* .006)	1.02 (0.99-1.04; *P =* .148)
Sex				
Female	59 (64.8)	32 (35.2)	-	-
Male	283 (68.2)	132 (31.8)	0.86 (0.54-1.40; *P =* .536)	0.47 (0.26-0.85; *P =* .013)
Baseline ALC, × 10^3^/μL				
Mean (SD)	1.7 (0.6)	1.4 (0.5)	0.36 (0.24-0.51; *P <* .001)	0.35 (0.23-0.51; *P <* .001)
PTV, cm^3^				
Mean (SD)	507.5 (223.2)	633.1 (238.2)	1.00 (1.00-1.00; *P <* .001)	1.00 (1.00-1.00; *P <* .001)
Radiation modality, no. (%)				
IMRT	147 (58.1)	106 (41.9)	-	-
PBT	195 (77.1)	58 (22.9)	0.41 (0.28-0.60; *P <* .001)	0.35 (0.22-0.53; *P <* .001)
ECOG status at trial entry, no. (%)				
1 and 2	137 (72.5)	52 (27.5)	-	-
0	205 (64.7)	112 (35.3)	1.44 (0.97-2.14; *P =* .070)	1.21 (0.77-1.91; *P =* .410)
Tumor location, no. (%)				
Upper-middle	56 (77.8)	16 (22.2)	-	-
Lower	286 (65.9)	148 (34.1)	1.81 (1.03-3.36; *P =* .048)	2.11 (0.94-4.91; *P =* .074)
Disease stage, no. (%)				
I	27 (84.4)	5 (15.6)	-	-
II	109 (72.7)	41 (27.3)	2.03 (0.79-6.30; *P =* .173)	2.32 (0.82-7.69; *P =* .134)
III	206 (63.6)	118 (36.4)	3.09 (1.26-9.32; *P =* .024)	3.13 (1.15-10.17; *P =* .037)
No. of concurrent chemotherapy cycles				
Mean (SD)	5.0 (0.5)	5.0 (0.6)	0.88 (0.63-1.22; *P =* .435)	0.80 (0.56-1.14; *P =* .203)
Barrett's syndrome, no. (%)				
No	325 (67.3)	158 (32.7)	-	-
Yes	17 (73.9)	6 (26.1)	0.73 (0.26-1.79; *P =* .509)	0.94 (0.30-2.69; *P =* .911)
Histology, no. (%)				
Adenocarcinoma	279 (66.6)	140 (33.4)	-	-
SCC	63 (72.4)	24 (27.6)	0.76 (0.45-1.25; *P =* .292)	0.91 (0.43-1.89; *P =* .793)
If patient received surgery, no. (%)				
No	146 (62.1)	89 (37.9)	-	-
Yes	196 (72.3)	75 (27.7)	0.63 (0.43-0.91; *P =* .015)	0.61 (0.39-0.97; *P =* .036)

*Abbreviations*: ALC = absolute lymphocyte count; ECOG = Eastern Cooperative Oncology Group performance status; IMRT = intensity-modulated (photon) radiation therapy; OR = odds ratio; PBT = proton beam therapy; PTV = planning target volume; SCC = squamous cell carcinoma.

### Comparison of OS by RIL and radiation modality

**Figure 1:**
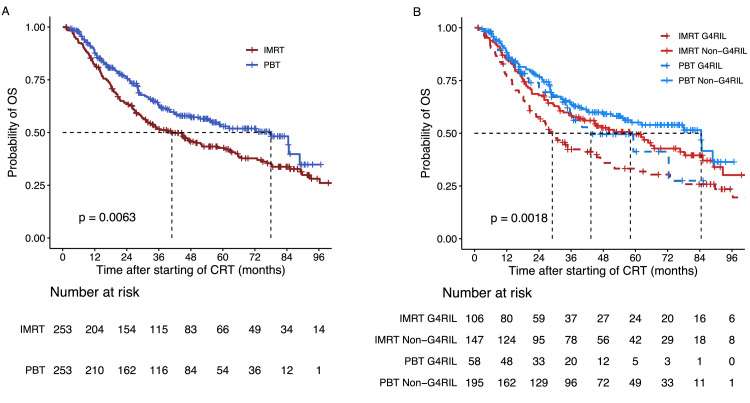
Propensity-matched cohort Kaplan-Meier overall survival (OS) curves by: A. radiation modality; B. radiation modality and occurrence of grade 4 radiation-induced lymphopenia (G4RIL). *Abbreviations*: CRT = chemoradiation therapy; IMRT = intensity-modulated radiation therapy; PBT = proton beam therapy.

Log-rank tests showed that OS varied significantly by G4RIL incidence (*P* = .0018) and radiation modality (*P* = .0063) (Fig. 1). Median OS time was 40.9 months for the IMRT group (95% CI, 31.4-58.1) versus 78.0 months for the PBT group (95% CI, 54.8 to not reached) (Fig. 1A). The corresponding 3-year OS rates were 51.66% for IMRT (95% CI, 45.72%-58.37%) and 63.10% for PBT (95% CI, 56.98%-69.89%) (Fig. 1B). Compared with patients receiving IMRT, patients receiving PBT had significantly prolonged OS in both univariate analysis (HR, 0.71; 95% CI, 0.55-0.91; *P* = .007) and multiple regression analysis (HR, 0.71; 95% CI, 0.55-0.93; *P* = .011) (Table 4). The development of G4RIL was significantly associated with a shorter median OS (G4RIL 34.7 months [95% CI, 27.8-47.5] vs non-G4RIL 65.7 months [95% CI, 51.5-85.1]) and lower 3-year OS rate (G4RIL 47.78% [95% CI, 40.31%-56.63%] vs non-G4RIL 61.59% [95% CI, 56.40%-67.26%]). PBT patients without G4RIL had the longest median OS time (84.5 months [95% CI, 54.8 to not reached]) compared with the other 3 subgroups (G4RIL PBT 43.4 months [95% CI, 34.0 to not reached]; non-G4RIL IMRT 58.1 months [95% CI, 36.5-85.1]; and G4RIL IMRT 29.0 months [95% CI, 23.0-35.6]) (Fig. 1B).

**Table 4:** Hazard ratios for overall survival for the propensity-matched cohort (N = 506)

Covariate	Value or n (%)	HR (univariable)	HR (multivariable)
Age, y			
Mean (SD)	65.0 (9.9)	1.02 (1.01-1.03; *P =* .007)	1.01 (1.00-1.03; *P =* .175)
Sex, no. (%)			
Female	91 (18.0)	-	-
Male	415 (82.0)	1.33 (0.95-1.88; *P =* .098)	1.44 (0.98-2.12; *P =* .064)
Baseline ALC, × 10^3^/μL			
Mean (SD)	1.6 (0.6)	1.01 (0.82-1.23; *P =* .958)	1.15 (0.93-1.42; *P =* .209)
PTV, cm^3^			
Mean (SD)	548.2 (235.4)	1.00 (1.00-1.00; *P =* .005)	1.00 (1.00-1.00; *P =* .172)
Radiation modality, no. (%)			
IMRT	253 (50.0)	-	-
PBT	253 (50.0)	0.71 (0.55-0.91; *P =* .007)	0.68 (0.53-0.89; *P =* .005)
ECOG status at trial entry, no. (%)			
1 and 2	189 (37.4)	-	-
0	317 (62.6)	1.13 (0.87-1.46; *P =* .351)	1.01 (0.77-1.32; *P =* .963)
Tumor location, no. (%)			
Upper-middle	72 (14.2)	-	-
Lower	434 (85.8)	0.73 (0.52-1.03; *P =* .070)	0.83 (0.53-1.29; *P =* .410)
Disease stage, no. (%)			
I	32 (6.3)	-	-
II	150 (29.6)	1.68 (0.84-3.37; *P =* .145)	1.76 (0.86-3.58; *P =* .119)
III	324 (64.0)	2.72 (1.39-5.31; *P =* .003)	2.77 (1.38-5.58; *P =* .004)
No. of concurrent chemotherapy cycles			
Mean (SD)	5.0 (0.6)	1.00 (0.77-1.31; *P =* .978)	0.95 (0.72-1.24; *P =* .688)
Barrett's syndrome, no. (%)			
No	483 (95.5)	-	-
Yes	23 (4.5)	0.82 (0.43-1.54; *P =* .528)	1.09 (0.57-2.09; *P =* .788)
G4RIL, no. (%)			
No	342 (67.6)	-	-
Yes	164 (32.4)	1.49 (1.16-1.92; *P =* .002)	1.22 (0.91-1.62; *P =* .181)
Histology, no. (%)			
Adenocarcinoma	419 (82.8)	-	-
SCC	87 (17.2)	1.25 (0.91-1.72; *P =* .163)	1.24 (0.82-1.88; *P =* .301)
If patient received surgery, no. (%)			
No	235 (46.4)	-	-
Yes	271 (53.6)	0.62 (0.49-0.80; *P <* .001)	0.62 (0.47-0.82; *P =* .001)

*Abbreviations*: ALC = absolute lymphocyte count; ECOG = Eastern Cooperative Oncology Group performance status; G4RIL = grade 4 radiation-induced lymphopenia; HR = hazard ratio; IMRT = intensity-modulated (photon) radiation therapy; PBT = proton beam therapy; PTV = planning target volume; SCC = squamous cell carcinoma.

### Survival mediation analysis

**Figure 2:**
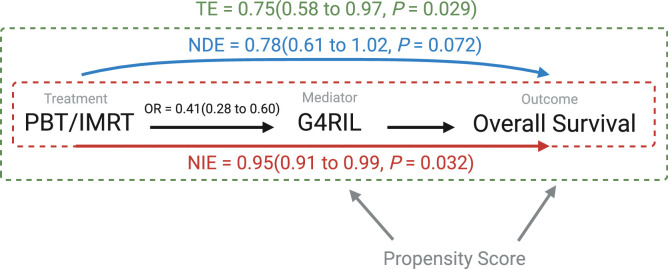
Causal diagram of mediation analysis relating radiation modality to the mediator (grade 4 radiation-induced lymphopenia [G4RIL]) and endpoint (overall survival) while controlling for selection bias by using propensity scoring. Hazard ratios are shown along each path. *Abbreviations*: IMRT = intensity-modulated radiation therapy; NDE = natural direct effect; NIE = natural indirect effect; OR = odds ratio; PBT = proton beam therapy; TE = total effect of G4RIL. Regression models included the propensity score to adjust treatment effects estimates for selection bias.

Mediation analysis was used to decompose the total effect of radiation modality on OS into (1) an indirect effect mediated through G4RIL and (2) the direct effect of modality on OS, where (1) defines the extent of OS benefit from PBT that is attributable to G4RIL risk reduction, and (2) quantifies the extent to which OS would have been improved for PBT if the risk of G4RIL from PBT was held identical to that from IMRT. The indirect effect of HR for OS was statistically significant at 0.95 (95% CI, 0.91-0.99; *P* = .032), as shown in Fig. 2. Therefore, the extent of G4RIL risk reduction associated with PBT contributed to a 5% reduction in the relative rate of death for patients receiving PBT compared with patients receiving IMRT. This corresponds to an estimated 2.37 months (95% CI, 1.89-2.99) of prolonged median survival time for PBT attributable to the lymphocyte-sparing effect compared with IMRT. As calculated by the method of Li et al,**^15^** approximately 14.5% of the total effect of radiation modality on OS was mediated through G4RIL. The unmediated G4RIL direct effect HR for OS was not statistically significant at 0.79 (95% CI, 0.61-1.02; *P* = .072). In other words, if the risk of G4RIL for PBT were identical to that of IMRT, then a statistically significant improvement in survival for PBT would not have been evident for this cohort.

### Surgical subgroup analysis

Mediation analyses stratified by surgery versus no surgery after chemoRT were also carried out. In subgroup analyses, the G4RIL-mediated effect of modality on OS remained in the same direction for both surgery and nonsurgery patients, with corresponding indirect effect HRs of 0.96 (95% CI, 0.91-1.01; *P* = .1184) and 0.98 (95% CI, 0.92-1.04; *P* = .5074), respectively.

## Discussion

Mediation analysis interrogates and compares chains of relations, whereby an antecedent variable affects a mediating variable, which then affects the endpoint. This study is the first to ascertain if radiation modality is a causal determinant of OS for patients with EC and to understand if G4RIL is a causal mediator of OS. Propensity-scoring techniques were applied to the retrospectively observed data to adjust for selection bias using measured confounders. Patients with EC receiving PBT in the matched subset experienced both a reduced risk of G4RIL (*P* < .001) and prolonged OS relative to patients receiving IMRT (*P* = .0063). Mediation analysis estimated that the 2.44-fold reduction in odds of G4RIL associated with PBT yielded a 5% reduction in the relative rate of death for patients receiving PBT. Consequently, management of radiation-induced immunosuppression during and after RT may be critical for improving the clinical care of patients with EC receiving concurrent chemoradiotherapy.

Our study results not only align with previous research indicating that G4RIL and radiation modality significantly impact OS in patients with advanced EC but also further extend statistical inference to directly estimate the causal effect of G4RIL risk reduction on survival. These results establish G4RIL as a causal mediator for survival in EC, which, to our knowledge, has not been reported previously.

Currently, RIL is generally ignored in clinical practice, and little attention is paid to mitigating its occurrence or severity. However, this paradigm is starting to change. In light of the increased use of immune checkpoint inhibitors after RT, the benefit of which may be abrogated for patients with severe lymphopenia, several options may be considered to adapt clinical RT practice to mitigate the incidence of severe lymphopenia. For example, several studies have shown that the radiation dose to large vessels and bone marrow^16^^,^^17^ strongly correlates with the development of RIL, indicating that purpose-driven dose optimization could help mitigate its incidence. The time factor is also important; both shorter fractionation regimens and higher dose rates^18^^,^^19^ have been proposed to mitigate lymphocyte depletion. Finally, several models for predicting patient-specific risk of RIL have been developed,^20^ which could be used to select high-risk patients for more conformal RT modalities such as PBT. Such models take into account each patient's baseline characteristics, such as age, disease stage, tumor location and volume, adjuvant and concurrent therapies, comorbidities, and others, as well as the dosimetric features. The prediction accuracy of these models is improved further if one or more lymphocyte count measurements are available at the beginning of treatments after the first few fractions.^21^ Such models can be useful for selecting treatment modality (eg, protons or photons), identifying dosimetric features that could be constrained further for optimization of dose distributions, and suggesting fractionation strategies that may lead to reduced RIL risk.

We should note further that the work reported here and in prior modeling publications was based on a PBT group that had been treated entirely with PSPT. Intensity-modulated proton therapy (IMPT), with its ability to control the intensities of small subdivisions of beams (“beamlets”) of a discrete sequence of energies, is capable of producing far more compact dose distributions, which can spare normal tissues, including immune organs at risk, to a considerably greater degree. Explicit incorporation of constraints on dosimetric determinants of RIL risk for IMPT optimization may further mitigate the risk.

This study had several limitations. For example, while the findings from the mediation analysis were directionally consistent, they lacked statistical significance in subgroup analyses stratified by surgical resection status. These subgroups were insufficiently powered for mediation analysis. Moreover, accurate interpretations of causal mediation models require control of confounders. In this study, potential confounders were controlled by propensity score analyses that evaluated all accessible covariates in the database. However, the reliance on retrospective data introduces potential biases related to the completeness and accuracy of records. The nature of the retrospective study means that we cannot infer the extent to which unmeasured confounders may have biased our conclusions. This limitation can be overcome only by prospective design with randomized intervention assignment. However, it is important to note that our data set is larger than those used in many previous studies, which enhances the robustness of our results. This study was conducted at a single tertiary care center, which may limit the generalizability of our results. Validation of our findings will be pursued as a part of future clinical trials, including the ongoing multicenter, nationally randomized controlled phase 3 NRG Oncology trial.^8^

## Conclusions

The findings reported here reinforce previous findings that severe immunosuppression (G4RIL) is prevalent among patients with EC treated with protons and photons. Moreover, severe RIL is associated with diminished OS. However, the incidence of G4RIL is significantly reduced, and correspondingly, survival is improved among patients treated with PBT. Our mediation analysis demonstrated that G4RIL is a causal determinant of OS and that differences in G4RIL after proton versus photon therapy augment the statistical significance of survival benefits from PBT. We should recognize that all patients in the PBT cohort were treated with PSPT and expect the advantage of protons over photons to increase with the use of IMPT, which is rapidly becoming the dominant proton therapy technique.

## Disclosures

Steven H. Lin discloses grant funding from Beyond Spring Pharmaceuticals, Nektar Therapeutics, STCube Pharmaceuticals, and IntraOp Corporation, serving on the advisory board for Beyond Spring Pharmaceuticals, STCube Pharmaceuticals, and AstraZeneca, and being a consultant for XRAD Therapeutics. Brian P. Hobbs discloses serving on the advisory board for CSL Behring and Telperian. All other authors: None.

## Appendix Supplementary materials

Mediation Model Supp Fig S1_ Revised.docx Image, application 1

## Footnotes

Supplementary material associated with this article can be found in the online version at doi:10.1016/j.adro.2024.101579.
