# Supplementary material for: Radiation-Induced Lymphopenia is a Causal Mediator of Survival After Chemoradiation Therapy for Esophagus Cancer
Source: Adv Radiat Oncol. 2024 Jul 26;9(10):101579. doi: 10.1016/j.adro.2024.101579 (PMC11382310; doi:10.1016/j.adro.2024.101579)
Supplement: Mediation Model Supp Fig S1_ Revised.docx [file mmc1.docx]

**Supplementary Figure E1.** Forest plot of odds ratio for propensity score analysis in the overall cohort and the matched cohort. PTV/100 is per 100 cm^3^ of planning target volume; Age/10 is per 10 years of age.
